# Combination of Tertiary Lymphoid Structure and Neutrophil-to-Lymphocyte Ratio Predicts Survival in Patients With Hepatocellular Carcinoma

**DOI:** 10.3389/fimmu.2021.788640

**Published:** 2022-01-13

**Authors:** Shaodi Wen, Yuzhong Chen, Chupeng Hu, Xiaoyue Du, Jingwei Xia, Xin Wang, Wei Zhu, Qingbo Wang, Miaolin Zhu, Yun Chen, Bo Shen

**Affiliations:** ^1^ The Affiliated Cancer Hospital of Nanjing Medical University, Jiangsu Cancer Hospital, Jiangsu Institute of Cancer Research, Nanjing, China; ^2^ Key Laboratory of Microenvironment and Major Diseases, Department of Immunology, Nanjing Medical University, Nanjing, China; ^3^ School of Medicine, Jiangsu University, Zhenjiang, China; ^4^ Department of Chemotherapy, The Second Hospital of Nanjing, Nanjing University of Chinese Medicine, Nanjing, China; ^5^ Jiangsu Key Lab of Cancer Biomarkers, Prevention and Treatment, Collaborative Innovation Center for Cancer Personalized Medicine, Nanjing Medical University, Nanjing, China

**Keywords:** hepatocellular carcinoma, tertiary lymphoid structure, neutrophil-to-lymphocyte ratio (NLR), overall survival, inflammation

## Abstract

**Background:**

Hepatocellular carcinoma (HCC) is the most common pathological type of primary liver cancer. The lack of prognosis indicators is one of the challenges in HCC. In this study, we investigated the combination of tertiary lymphoid structure (TLS) and several systemic inflammation parameters as a prognosis indicator for HCC.

**Materials and Methods:**

We retrospectively recruited 126 postoperative patients with primary HCC. The paraffin section was collected for TLS density assessment. In addition, we collected the systemic inflammation parameters from peripheral blood samples. We evaluated the prognostic values of those parameters on overall survival (OS) using Kaplan-Meier curves, univariate and multivariate Cox regression. Last, we plotted a nomogram to predict the survival of HCC patients.

**Results:**

We first found TLS density was positively correlated with HCC patients’ survival (HR=0.16, 95% CI: 0.06 − 0.39, *p* < 0.0001), but the power of TLS density for survival prediction was found to be limited (AUC=0.776, 95% CI:0.772 − 0.806). Thus, we further introduced several systemic inflammation parameters for survival analysis, we found neutrophil-to-lymphocyte ratio (NLR) was positively associated with OS in univariate Cox regression analysis. However, the combination of TLS density and NLR better predicts patient’s survival (AUC=0.800, 95% CI: 0.698-0.902, *p* < 0.001) compared with using any single indicator alone. Last, we incorporated TLS density, NLR, and other parameters into the nomogram to provide a reproducible approach for survival prediction in HCC clinical practice.

**Conclusion:**

The combination of TLS density and NLR was shown to be a good predictor of HCC patient survival. It also provides a novel direction for the evaluation of immunotherapies in HCC.

## Introduction

Primary liver cancer is the third leading cause of cancer-related deaths globally, accounting for approximately 782,500 deaths annually. Hepatocellular carcinoma (HCC) is the most predominant type of primary liver cancer, comprising about 90% of cases (1, 2). The incidence of HCC is increasing, from 14 million cases worldwide in 2012 to an estimated 22 million cases in 2030 (3). Systemic therapy options for HCC are limited, and early-stage HCC was also prone to recurrence and metastasis after surgery. The complex tumor microenvironment of HCC was believed to contribute to the difficulty of treating patients with HCC (4). Many predictive markers for prognosis and postoperative recurrence and metastasis in patients with HCC have been proposed. However, none of them was proved to be credible.

In recent years, tertiary lymphoid structures (TLSs) have been identified as ectopic lymphoid organs in non-lymphoid tissues at sites of chronic inflammation, including tumors. TLSs are existed in tumors in different states of maturation, culminating in the formation of germinal centers. TLSs represent the local immune infiltration status in the tumor microenvironment. The density of TLS in the paraneoplastic tissues is strongly associated with patient prognosis. The presence of TLSs has been shown to be a protective predictor of prognosis in tumors such as breast (5, 6), lung (7, 8), colon (9), malignant melanoma (10), ovarian (11), gastric (12), pancreatic (13), and squamous head and neck cancers (14). TLSs were correlated with the HCC patients’ survival; however, the prognostic value of TLSs remains unclear. Most studies found that the presence of TLSs was a positive prognostic factor for HCC patients (15, 16), while Firkin et al. concluded that the presence of TLSs was a poor prognostic factor (17). They have analyzed the relationship between TLS and the prognosis of HCC patients at several levels, including clinical data and mouse models, and have explored possible mechanisms. Validation at the TLS protein and gene levels concluded that TLS is associated with poor prognosis in HCC patients. Therefore, the relationship between TLSs and HCC patient survival warrants further validation.

Several systemic inflammation parameters are used to predict the HCC patients’ survival, such as neutrophil to lymphocyte ratio (18) (NLR), platelet to lymphocyte ratio (19) (PLR), lymphocyte to monocyte ratio (20) (LMR), and systemic immune inflammation index (21) (SII), which is considered as a predictor of multiple therapies in HCC patients (22–25). Among those, the NLR is the most studied indicator of systemic inflammation, and it also represents to some extent the systemic immune status (26, 27). It can be used as a meaningful and straightforward marker for assessing systemic imbalances of myeloid immunosuppression, as it is an inexpensive and easily measured marker of systemic inflammation that is associated with predictive prognosis in a variety of cancers, including liver, lung, and rectal cancers. Some retrospective clinical studies have shown that a negative correlation between NLR and tumor prognosis. Besides, immunotherapy drugs have increasingly become the standard of care for various cancer, but only a minority of patients respond to immunotherapy. Studies have shown that NLR can predict the efficacy of immunotherapy in lung cancer (28) and HCC (29) by identifying those who responded to immunotherapy and avoiding the adverse consequences of continuing treatment in those who fail to respond to immunotherapy. In addition, PLR, LMR and SII can also predict the prognosis of HCC patients. Elevated PLR was found to be associated with poor overall survival (30, 31). Low LMR is a risk factor for disease-free survival (32). However, neither of them is the best way to predict the survival of HCC patients.

In this paper, the relationship between the tumor microenvironment and patient prognosis in patients with hepatocellular carcinoma was explored to guide clinicians in the comprehensive prognosis of the immune status in patients with HCC after surgery and to identify patients with a potentially poor prognosis at an early stage. Specifically, the role of TLSs in tissue sections, TLS density, systemic inflammation parameters in the prognosis of HCC patients was investigated using survival analysis in a retrospective cohort.

## Materials and Methods

### Study Population

We retrospectively enrolled patients with HCC diagnosed by surgical pathology at Jiangsu Cancer Hospital, Nanjing Second Hospital, and Jiangsu Provincial People’s Hospital from April 2013 to August 2019. All patients were treated with radical surgery and followed up until December 30, 2020. The following patients were excluded: I. with comorbidity of severe diabetes mellitus, heart failure, liver, and/or kidney failure; II. have a history of schizophrenia; III. have a history of other malignancies or postoperative detection of metastatic liver tumors; IV. dead during surgery in hospital; V. have another organ removed during surgery; VI. special populations, such as pregnant and lactating women. In total, we enrolled 126 patients and divided them into training and validation sets in the ratio of 2:1. This study has been approved by the institutional review board.

### Clinical Characteristics and Laboratory Parameters

The clinical characteristics and laboratory parameters data of each patient were obtained from the electronic medical records. Clinical characteristics included age, gender, Eastern Cooperative Oncology Group performance status (ECOG PS), Barcelona clinic liver cancer (BCLC), Child-Pugh class, histology, cause of hepatitis and their survival time, etc. Other laboratory parameters were collected seven days prior to the initiation of surgery, including a complete peripheral blood count. Systemic inflammation parameters, including NLR, PLR, LMR and SII, were calculated. NLR (absolute neutrophil count/absolute lymphocyte count), PLR (absolute platelet count/absolute lymphocyte count), LMR (absolute lymphocyte count/monocyte), SII, (neutrophil count*platelet count/lymphocyte count). Tumor markers and liver function indicators were also collected within 7 days before surgery.

### Immunohistochemistry

All specimens were prepared into 5 µm formalin-fixed paraffin-embedded (FFPE) sections. After dewaxing the xylene clear, the sections were deparaffinized *via* a series of decreasing concentrations of ethanol. Endogenous peroxidase activity was blocked by incubation in a 3% methanol solution of H_2_O_2_. Antigenic epitopes were unmasked in a decloaking chamber using citrate buffer (10 mM sodium citrate and 0.05% Tween 20, pH=6). The sections were then washed in deionized water, rinsed in PBS, blocked at room temperature with 5% BSA in PBS for 30 min, and incubated with primary antibodies in a humidified chamber at 4 °C overnight. After washing, the sections were incubated with anti-rabbit/mouse IgG monoclonal antibodies at room temperature for 1 hour. Staining was performed using Diaminobenzidine, followed by counterstaining with hematoxylin.

### Immunofluorescence

For immunofluorescence staining, all specimens were prepared into 5 µm FFPE sections. For CD20 staining, primary antibodies with goat anti-rabbit CD20 (Abcam, Cambridge, UK, 1:500). For DC-Lamp staining, primary antibodies with rat anti-rabbit DC-Lamp (SIGMA, Ronkonkoma, America, 1:100). For CD21 staining, rat anti-rabbit CD21 (Abcam, Cambridge, UK, 1:500) antibody was used and rabbit anti-rabbit CD23 (Abcam, Cambridge, UK, 1:100) was used. Sections were then incubated in the fluorescent-conjugated secondary antibodies (Thermo Scientific) and analyzed under a microscope.

### Tertiary Lymphoid Structures

The number of dense lymphocytic aggregates has been quantified per 10x high-power field (HPF) in all tumor-containing haematoxylin and eosin (H&E)-stained diagnostic sections of our cohort. TLS density had been calculated as the number of TLS per mm2 in peritumoral regions. A patient had considered as germinal centers positive (GC-positive) if at least one TLS showed the characteristic morphology of proliferating centroblasts. All pathology sections are evaluated by two pathologists individually, with different opinions discussed and the final decision made by the senior doctor.

### Calculation of Density of Tertiary Lymphatic Structures

The TLS we calculated was the density of TLS observed in the intertumoral and peritumoral 5 mm locations. The Olympus microscope BX51 was used, which was marked with an eyepiece with a field of view of 22. TLS were counted per 10X field. Diameter (d) = 0.22 mm, S = π1/4d^2^ = 3.8mm^2^. TLS density = Total of 5 random views/5/S, as described previously (7, 15).

### Statistical Analyses

Statistical analyses were performed using RStudio (version 3.6.3) and SPSS (version 26.0). Cutoff points of TLS density, PLR, LMR, SII and NLR were determined using the receiver operating characteristics (ROC) curve analysis. The ROC curve was constructed using survival status and each indicator, the best cut-off was when the AUC was the maximum. Kaplan–Meier curves were plotted for TLS density, NLR, PLR, LMR, and SII, respectively. Cox proportional hazards regression analysis was conducted to identify indicators associated with overall survival (OS). Variables with a *p* value less than 0.05 in the univariate Cox regression analysis were included in a multivariate analysis to identify which variables were significantly associated with survival. Nomograms to predict survival probability at 24- and 60-months for HCC patients were constructed for each identified predictive factor. Each nomogram was also validated internally using a bootstrap method with 1000 resamples. Concordance index (C-index) values were calculated to evaluate the predictive ability of each factor, with 0.5 indicating random chance and closer to 1.0 indicating a better ability to correctly discriminate the outcome. Calibration curves were used to assess the correlation between actual outcomes and predicted probabilities.

## Results

### Patients’ Characteristics

We retrospectively enrolled 126 postoperative patients with primary HCC whose baseline characteristics are shown in **Table 1** and **Supplement Tables 2A, B**. All included populations were patients with early-stage surgically operable HCC who did not receive any preoperative anti-tumor therapy. The median follow-up time for the enrolled patients was 44 months in the training set (95%CI: 37.162 - 50.838 months). The flow chart of tissue sections and liquid biopsy indicators collection was shown in **Supplement Figure 1**. Variables that conform to the normal distribution are expressed as mean ± standard deviation (SD). Variables that have skewed distribution are expressed using the median and interquartile ranges (IQRs). Details were shown in **Supplement Table 1**.

**Table 1:** Associations between clinical factors and different indicators in patients with hepatocellular carcinoma.

	NLR High	NLR Low	P-value	TLS High	TLS Low	P-value
	(N = 34)	(N = 92)		(N = 61)	(N = 65)	
**Gender**						
man	30 (88.2%)	75 (81.5%)	0.53	52 (85.2%)	53 (81.5%)	0.75
woman	4 (11.8%)	17 (18.5%)		9 (14.8%)	12 (18.5%)	
**Age(years)**						
age<54	20 (58.8%)	30 (32.6%)	0.0137	24 (39.3%)	26 (40.0%)	1
age≥54	14 (41.2%)	62(67.4%)		37 (60.7%)	39 (60.0%)	
**ECOG PS**						
0/1	28 (82.3%)	81 (88.0%)	0.0218	56 (91.1%)	53 (70.0%)	0.144
2	6 (17.6%)	11 (12.0%)		5 (8.2%)	12 (30.0%)	
**Child Pugh class**						
A	26 (76.5%)	89 (96.7%)	0.00127	55 (90.2%)	60 (92.3%)	0.912
B	8 (23.5%)	3 (3.3%)		6 (9.8%)	5 (7.7%)	
**BCLC**						
A	24 (70.6%)	67 (72.8%)	0.856	49 (80.3%)	42 (64.6%)	0.112
B	2 (5.9%)	7 (7.6%)		4 (6.6%)	5 (7.7%)	
C	8 (23.5%)	18 (19.6%)		8 (13.1%)	18 (27.7%)	
**Alb (g/L)**						
>44.20	22 (64.7%)	48 (52.2%)	0.292	34 (55.7%)	36 (55.4%)	1
≤44.20	12 (35.3%)	44 (47.8%)		27 (44.3%)	29 (44.6%)	
**Cause of hepatitis**						
HAV	1 (2.9%)	1 (1.1%)	0.386	1 (1.6%)	1 (1.5%)	0.978
HBV	20 (58.8%)	65 (70.7%)		40 (65.6%)	45(69.2%)	
HCV	0 (0%)	2 (2.2%)		1 (1.6%)	1 (1.5%)	
Unknown	13 (38.2%)	24 (26.1%)		19 (31.1%)	18 (27.7%)	
**AFP (ng/ml)**						
>37.96	15 (44.1%)	46 (50.0%)	0.700	29 (47.5%)	32 (49.2%)	0.991
≤37.96	19 (55.9%)	46 (50.0%)		32 (52.5%)	23 (50.8%)	
**CEA (ng/ml)**						
>2.16	17 (50.0%)	52 (56.5%)	0.652	30 (49.2%)	39 (60.0%)	0.298
≤2.16	17 (50.0%)	40 (43.5%)		31 (50.8%)	26 (40.0%)	
**TB(μmol/L)**						
>14.80	22 (64.7%)	40(43.5%)	0.055	23 (37.7%)	39 (60.0%)	0.0202
≤14.80	12 (35.3%)	52 (56.5%)		28 (62.3%)	26 (40.0%)	
**PT(s)**						
>13.00	17 (50.0%)	31 (33.7%)	0.143	15 (24.6%)	33 (50.8%)	0.0045
≤13.00	17 (50.0%)	61 (66.3%)		46 (75.4%)	32 (49.2%)	
**APTT(s)**						
>30.10	18 (52.9%)	35 (38.0%)	0.193	18 (29.5%)	35 (53.8%)	0.00974
≤30.10	16 (47.1%)	57 (62.0%)		43 (70.5%)	30 (46.2%)	
**TT(s)**						
>18.40	13 (38.2%)	44 (47.8%)	0.448	31 (50.8%)	26 (40.0%)	0.298
≤18.40	21 (61.8%)	48 (52.2%)		30 (49.2%)	39 (60.0%)	
**GGT(U/L)**						
>52.20	24 (70.6%)	43 (46.7%)	0.0292	31 (50.8%)	36 (55.4%)	0.738
≤52.20	10(29.4%)	49(55.4%)		30 (49.2%)	29 (44.6%)	
**ALT(U/L)**						
>30.00	25 (73.5%)	39 (42.4%)	0.0037	33 (54.1%)	31 (47.7%)	0.589
≤30.00	9 (26.5%)	53 (57.6%)		28 (45.9%)	34 (52.3%)	
**AST(U/L)**						
>31.80	26 (76.6%)	41 (44.6%)	0.00284	33 (54.1%)	34 (52.3%)	0.738
≤31.80	8 (23.5%)	51(55.4%)		28 (45.9%)	31 (47.7%)	

Data were expressed as n (%) and median (interquartile range). ECOG PS Eastern Cooperative Oncology Group performance status, BCLC Barcelona clinic liver cancer, Alb albumin, HAV hepatitis A virus, HBV hepatitis B virus, HCV hepatitis C virus, AFP alpha-fetoprotein, CEA carcinoembryonic antigen, TB Total Bilirubin, PT prothrombin time, APTT activated partial thromboplastin time, TT thrombin time, GGT glutamyl transpeptidase, ALT alanine transaminase, AST aspartate transaminase.

### Identification of Tertiary Lymphoid Structure

**Figure 1:**
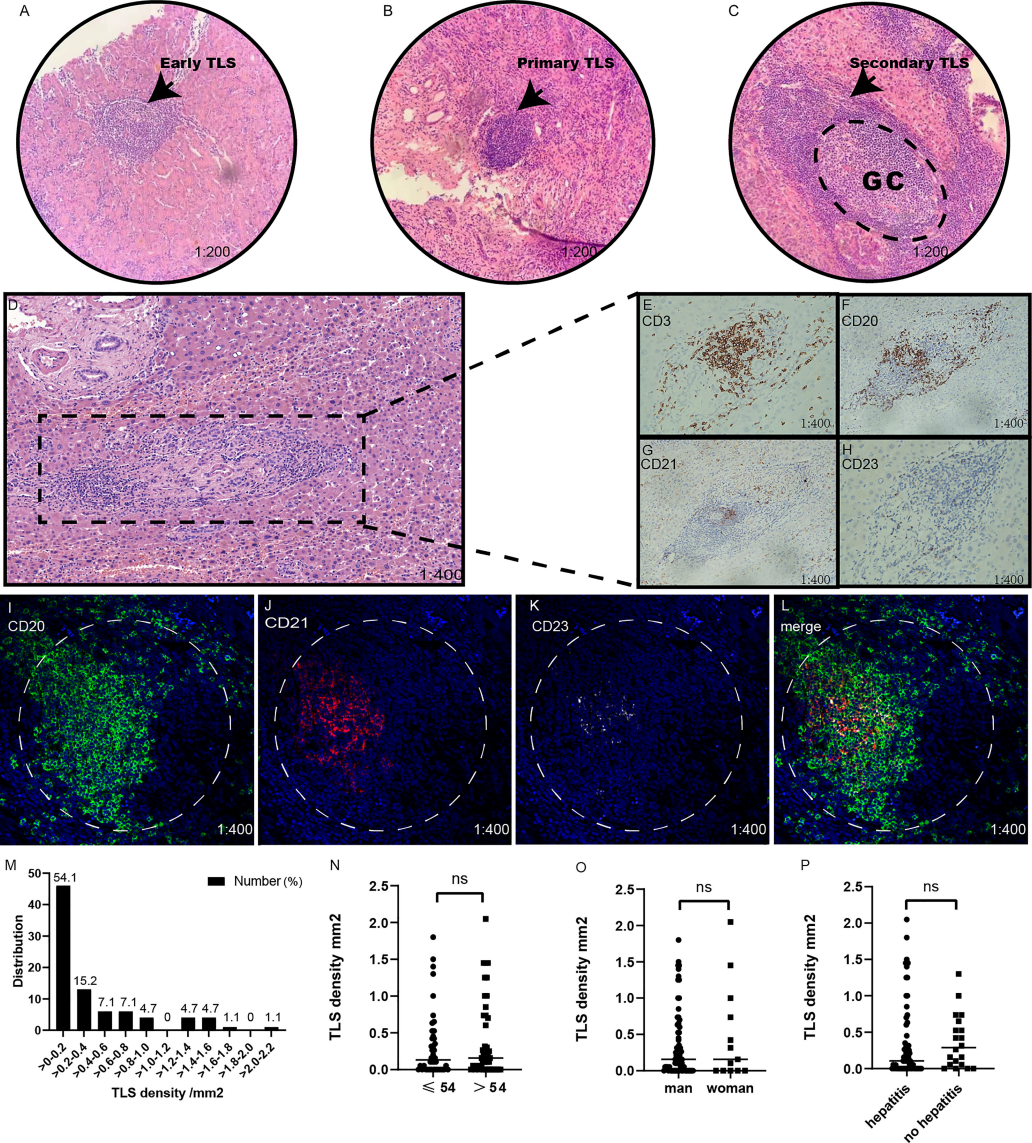
Identification and detection of tertiary lymphoid structures. **(A–D)**. H&E, early tertiary lymphoid structures for lymphocyte aggregation, **(B)** primary lymphoid structures; **(C)** secondary lymphoid structures, germinal centers positive (GC-positive), **(D)** secondary lymphoid structures with GC. **(D–H)**. the immunohistochemistry of. CD20^+^ B cells, CD21^+^ follicular helper T cells, and CD23^+^ germinal Centre scenters; **(I–L)** the co-stained immunofluorescence of CD20^+^, CD21^+^, and CD23^+^; **(L)** is merged by CD20^+^, CD21^+^, and CD23^+^’s merged. **(M)** The distribution of TLS density. **(N–P)** The relationship between age, gender and hepatitis. “ns” means no significance.

TLSs presented in many solid tumors and aggregated lymphocytes were considered as the typical structure of TLSs (33). The aggregation of CD20^+^ B cells was considered as the early feature of TLSs (7). And the TLSs with the infiltration of CD21^+^ follicular dendritic cells (FDC) were considered as primary TLSs. After that, with mature dendritic cells (mDC, CD23^+^), the TLSs were further described as 2^nd^ TLSs. From our HCC H&E-staining, we observed the presence of multistage maturation of TLS (from early TLSs, primary TLSs to secondary TLSs, and TLSs at different stages could be observed from the same section) (**Figures 1A–C**). To evaluate the TLS density, we quantified the numbers of TLSs in 5 random microscope bright light fields, and TLS density was calculated as the number of TLS per mm^2^ in peritumoral regions. The distribution of TLS density was presented in **Figure 1M**. Notably, more than 30% of HCC patients had a TLS density of 0 in over. Still, we did not find any difference in TLS density between different ages, genders, or hepatitis virus infection status were statistically significant (**Figures 1N–P**). After that, in our immunohistochemistry staining (IHC) with serial sections, we further confirmed the infiltrated immunocytes with cell-type-specific surface markers (**Figures 1D–H**). For further validation, we also performed immunofluorescence staining (IF), and observed CD20^+^, CD21^+^, and CD23^+^ at different stages of TLSs. In addition, we verified the presence of TLS using CD3, CD20 and DC-Lamp (**Figures 1I–L** and **Supplement Figures 2A-L**). In summary, we evaluated the TLS densities in HCC patients, and around 30% of the HCC patients failed to show the existence of TLSs.

### TLS Density and HCC Patient’s Survival

**Figure 2:**
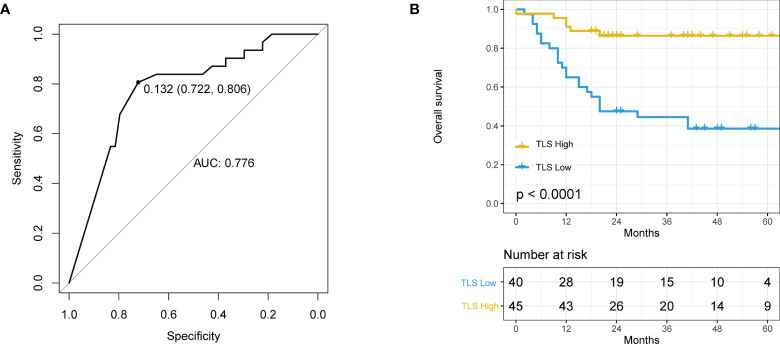
The relationship between tertiary lymphoid structure (TLS) and overall survival (OS) in hepatocellular carcinoma (HCC) patients. **(A)** Receiver operating characteristics (ROC) curve of TLS density and patient prognosis. The best cut-off is 0.132, and when using this point for segmentation, the sensitivity is 0.806 and the specificity is 0.772. **(B)** Kaplan–Meier curve using the best cutoff value of TLS density determined by A.

The best cutoff value for TLSs was determined by ROC curves, and the included population was accordingly divided into TLS^+^ and TLS^-^ groups. The best cutoff value for TLSs was 0.132 with an AUC value of 0.776 (95%CI 0.722-0.806, **Figure 2A**). The AUC (AUC=0.776, 95%CI:0.772−0.806) was limited even at the best cut-off point of TLS. The median overall survival for the TLS^-^ cohort was 20 months, while patients in the TLS^+^ group did not achieve 50% mortality. TLS density significantly associated with HCC patients’ survival (HR=0.16 95% CI: 0.06 − 0.39, *p* < 0.0001, **Figure 2B**).

### The Prognostic Value of Periphery Blood Systemic Inflammation Parameters in HCC Patients

**Figure 3:**
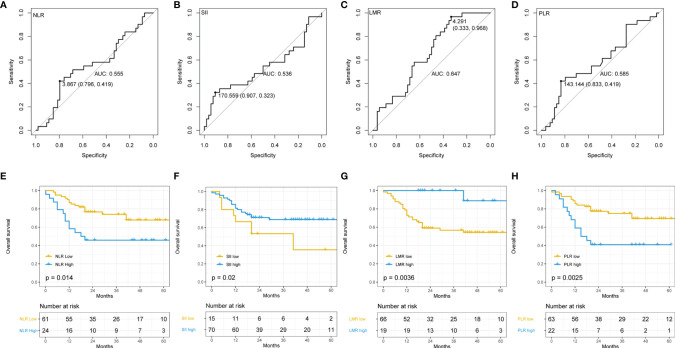
Relationship between indicators of inflammation in peripheral blood and overall survival (OS). **(A)** Receiver operating characteristics (ROC) curve of neutrophil to lymphocyte ratio (NLR) and patient prognosis; **(B)** ROC curve of systemic immune inflammation index (SII) and patient prognosis; **(C)** ROC curve of lymphocyte to monocyte ratio (LMR) and patient prognosis; **(D)** ROC curve of platelet to lymphocyte ratio (PLR) and patient prognosis; **(E)** Kaplan–Meier curve using the best cutoff value of NLR determined by **Figure 2A**; **(F)** Kaplan–Meier curve using the best cutoff value of SII determined by **Figure 2B**; **(G)** Kaplan–Meier curve using the best cutoff value of LMR determined by **Figure 2C**; **(H)** Kaplan–Meier curve using the best cutoff value of PLR determined by **Figure 2D**.

We evaluated the prognostic value of systemic inflammation parameters in HCC. ROC curves were introduced to determine the best cutoff values for each parameter. The best cutoff value for NLR, SII, LMR, and PLR the best were 3.867 (AUC=0.555, 95% CI: 0.796-0.419), 170.559 (AUC=0.536, 95% CI: 0.907,0.323), 4.291 (AUC=0.647, 95% CI: 0.333,0.968), and 144 (95% CI 0.833,0.419, AUC=0.585), respectively (**Figures 3A–D**). Median survival time was higher in patients with NLR^-^ (not achieved vs. 18 months, *p*=0.014, **Figure 3E**), SII^+^ (57 months vs. 43 months, *p*=0.020, **Figure 3F**), LMR^+^ (48 months vs. 37 months, *p*=0.0036, **Figure 3G**), PLR^-^ (48 months vs. 43 months, *p*=0.0025, **Figure 3H**). We also observed limited specificity and sensitivity for those parameters.

### Combining TLS and NLR to Predict Survival in HCC Patients

**Figure 4:**
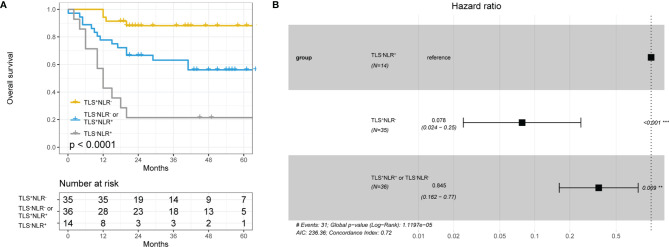
**(A)** Kaplan–Meier curve using the best cutoff value of lymphocyte ratio (NLR) determined by **Figures 2A**, **3A** group1 is patients in the TLS^+^NLR^-^ group, group2 is patients in the TLS^-^NLR^+^ or TLS^+^NLR^+^ group, and group3 is patients in the TLS^-^NLR^+^ group. **(B)** Hazard ratios and 95% CI in the three subgroups.

We found that TLS and several systemic inflammation parameters were correlated with HCC patients’ survival (**Figures 2B**, **3E–H**). In the univariate Cox regression, ECOG PS, TB, GGT, TLSs and NLR were significantly associated with OS. After including all those variables in the same model, only ECOG PS, TLS, and NLR were significantly associated with OS [**Table 2**, ECOG PS (HR=0.151,95%CI: 0.102-1.360, *p*=0.032), TLSs (HR=0.214, 95%CI:0.083-0.553, *p*=0.001), NLR (HR=2.578, 95%CI: 1.008-6.593, *p*=0.048)]. We performed an interaction analysis of TLS and NLR which found there was no interaction between them (*p*=0.878), and the interaction plot was shown in **Supplement Figure 3**. Therefore, we further create a new variable that combined TLSs and NLR to better predict the prognosis of HCC patients’ survival. Patients were divided into three groups: group 1 was patients with TLS^+^NLR^-^, group 2 was patients with TLS^+^NLR^+^ or TLS^-^NLR^-^ to indicate that patients with one positive poor prognostic factor, and group 3 was patients with TLS^-^NLR^+^. Median survival time did not reach in group 1, while it was 86 months (95%CI: 22.169-149.832 months) in group 2, and 12 months (95%CI: 8.371-15.629 months) in group 3. The combination of TLS density and NLR was significantly associated with OS (HRgroup2 = 0.078, 95% CI: 0.024 − 0.25, *p* = < 0.0001; HRgroup3 = 0.845, 95% CI: 0.162 − 0.77, *p* = < 0.0001). Kaplan–Meier curve and cox regression hazard ratios on OS were presented in **Figures 4A, B**. The combined prediction improves the limitations of TLS and NLR alone in predicting the prognosis of HCC patients’ survival, with an AUC value of 0.800 (95% CI 0.698-0.902, *p*<0.001, **Supplement Figure 4**).

**Table 2:** Univariate and Multivariate Cox regression analysis for OS.

	Overall Survival					
	Univariate			Multivariate		
	Hazard ratio	95%CI	*P*	Hazard ratio	95%CI	*P*
Age	0.992	0.961-1.024	0.618	–	–	–
Gender	1.220	0.426-3.495	0.712	–	–	–
ECOG PS						
2	1			1	–	–
0	0.052	0.018-0.150	**0.000**	0.151	0.027-0.849	**0.032**
1	0.218	0.098-0.484	**0.000**	0.372	0.102-1.360	0.135
Child-Pugh class	0.280	0.120-0.654	**0.003**	0.310	0.069-1.402	0.128
BCLC						
C	1			1	–	**-**
A	0.126	0.058-0.275	**0.000**	0.247	0.059-1.038	0.056
B	0.490	0.112-2.150	0.345	0.940	0.147-5.994	0.948
AFP	1.000	1.000-1.000	0.064	–	–	–
CEA	0.874	0.704-1.086	0.224	–	–	–
CA199	1.000	0.998-1.002	0.937	–	–	–
TB	1.008	1.003-1.013	**0.001**	1.001	0.996-0.985	0.529
ALT	1.000	0.998-1.002	0.783	–	–	–
AST	1.000	0.998-1.002	0.923	–	–	–
GGT	1.002	1.001-1.004	**0.000**	1.001	0.999-1.003	0.460
PT	1.135	0.987-1.306	0.077	–	–	–
APTT	1.031	0.992-1.072	0.125	–	–	–
TT	1.090	0.954-1.247	0.206	–	–	–
ANC	0.956	0.875-1.044	0.318	–	–	–
AMC	1.371	0.517-3.637	0.526	–	–	–
ALC	0.575	0.327-1.010	0.054	–	–	–
PLR^a^	0.998	0.992-1.003	0.373	–	–	–
LMR^b^	1.488	0.681-3.249	0.319	–	–	–
SII^c^	1.208	0.464-3.150	0.699	–	–	–
TLS^d^	0.161	0.065-0.396	**0.000**	0.214	0.083-0.553	**0.001**
NLR^e^	0.422	0.206-0.864	**0.018**	2.578	1.008-6.593	**0.048**

ECOG PS Eastern Cooperative Oncology Group performance status, BCLC Barcelona Clinic Liver Cancer, AFP alpha-fetoprotein, CEA carcinoembryonic antigen, TB Total Bilirubin, PT prothrombin time, APTT activated partial thromboplastin time, TT thrombin time, GGT glutamyl transpeptidase, ALT alanine transaminase, AST aspartate transaminase., ANC absolute neutrophil count, AMC absolute monocyte count, ALC Absolute lymphocyte count, PLR Platelet to lymphocyte ratio, LMR Lymphocyte to monocyte ratio, SII Systemic immune inflammation index, TLS tertiary lymphoid structures, NLR neutrophil-to-lymphocyte ratio, PLR platelet-lymphocyte ratio. ^a^Divided into PLR high and PLR low. ^b^Divided into LMR high and LMR low. ^c^Divided into SII high and SII low. ^d^Divided into TLS high and TLS low. ^e^Divided into NLR high and NLR low.

The bold values means statistically significant.

### Nomogram Provides Solid Survival Prediction

**Figure 5:**
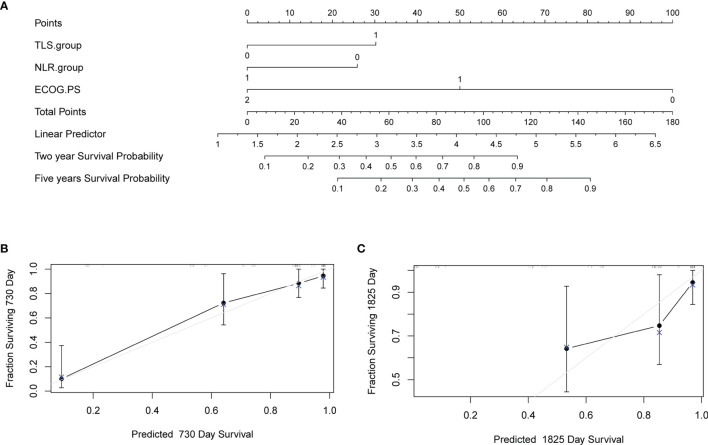
Nomogram to predict survival and the calibration curves of the Nomogram to predict survival. **(A)** Nomogram was developed based on three factors including baseline lymphocyte ratio (NLR), tertiary lymphoid structure (TLS), and Eastern Cooperative Oncology Group performance status (ECOG PS) to predict the probability of survival at 24- and 60-months. The probability could be obtained as a function of total points calculated as the sum of points for each specific variable. Points were assigned for each factor by drawing a line upward from the corresponding values to the ‘point’ line. The total sum of points added by each factor was plotted on the “total points” line. A line was drawn down to read the corresponding predictions of probability. Internal validation was performed by Bootstrap method with 1000 replicate samples. **(B)** A Calibration curves of a nomogram to predict survival at 24-months. **(C)** A Calibration curves of a nomogram to predict survival at 60-months.

**Figure 6:**
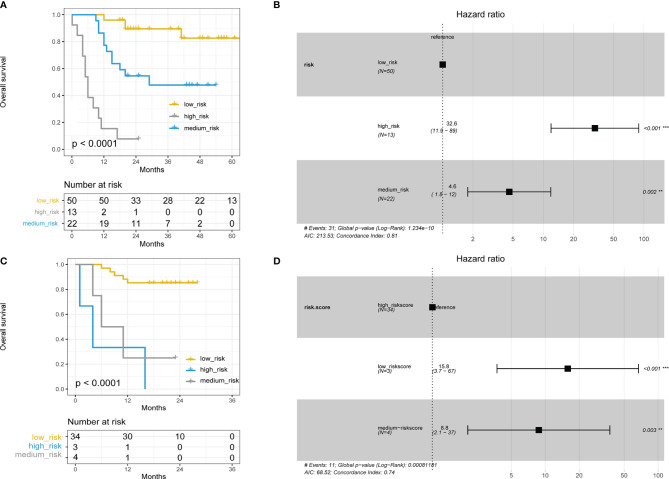
**(A)** Kaplan–Meier curve using the tertile of the model-predicted score (Training set). Patients were grouped into low-risk, medium-risk, and high-risk groups. **(B)** Hazard ratios and 95% CI in the three subgroups (Training set). **(C)** Kaplan–Meier curve using the tertile of the model-predicted score (Validation set). Patients were grouped into low-risk, medium-risk, and high-risk groups. **(D)** Hazard ratios and 95% CI in the three subgroups (Validation set).

A nomogram was constructed to predict survival based on the predictors identified in the multivariate Cox regression analyses. The nomogram was developed based on TLS, NLR, and ECOG PS to predict the probability of OS at 2 years and 5 years. A point scale score was assigned to each factor level and the factor scores were summed to obtain a total score, which helped to estimate the probability of survival at 2 and 5 years for each patient with HCC. The nomogram was internally validated with a C-index of 0.853 (95% CI: 0.560-0.854). Moreover, the calibration plot of the nomogram demonstrated a good consistency between the actual clinical results and the predicted outcomes (**Figure 5**). Then, survival curves were used to evaluate the nomogram’s discrimination power in predicting OS (**Figures 6A, B**). According to the tertile of the model-predicted score, patients were grouped into low-risk (HR= 32.6, 95% CI: 11.9 – 89.0, *p* < 0.001), medium-risk (HR= 4.6, 95% CI: 1.8 – 12.0, *p* < 0.002), and high-risk groups. We also performed an external validation of this model with a C-index of 0.876 (95% CI: 0.833-0.919). The validation set was grouped according to the risk scores derived from the training set and the survival curves were shown in **Figures 6C, D**.

## Discussion

In the current study, we assessed the immune infiltration status of HCC patients from the peripheral blood and primary tumor tissue levels. Our results suggest that both TLSs and NLR are good predictors of the prognosis in HCC patients, but limitations on survival prediction based on a single parameter were also observed. Therefore, we introduced a combination of clinical parameters (from primary tissue and peripheral blood) to better predict survival in HCC patients. We found that the combination of TLS, NLR, and ECOG PS could improve the survival prognosis of HCC patients, and therefore we designed a nomograph for clinicians that can easily help them to assess the prognosis for HCC patients after surgery.

TLS develops at sites of chronic inflammation and expands in response to pro-inflammatory cytokines. HCC is an example of an inflammation-driven cancer characterized by cirrhotic nodules with abundant TLS in non-tumor tissue (34). In a study of 82 HCC patients undergoing surgery for resection, expression of a 12-gene signature of TLSs and detection of TLSs in liver tissue adjacent to the tumor were found associated with an increased risk of late recurrence (17). However, Calderaro et al. reported in a total of 490 patients (combination of 2 different retrospective studies) the presence of true TLSs in the tumor region of HCC, but at a lower density than TLSs in the tumor-adjacent parenchyma. Compared with results obtained in non-tumor liver tissue, TLSs in the tumor core were negatively associated with the risk of early recurrence in both univariate and multivariate analysis. The risk of recurrence was also negatively correlated with the degree of TLSs maturation, with fully formed primary or secondary follicles providing better protection than lymphocyte aggregates alone (15). Our study assessed TLS density in paraneoplastic tissue, and the presence of TLSs in paraneoplastic tissue in our HCC cohort showed a correlation with better prognosis, which supported the Calderaro et al. findings. Firkin’s team explored TLSs mainly in the early stages of HCC patients, and TLSs were also observed to be associated with a poorer prognosis in tissues from human and mouse HCC models. This highlights the existence of distinct roles of TLSs in cancer, which may reflect alternative phenotypes of TLSs. That is, there are different functional roles for different TLSs.

Inflammatory responses play vital roles in the different stages of tumor development, including initiation, promotion, malignant conversion, invasion, and metastasis. Inflammation also affects immune surveillance and responses to therapy. Immune cells that infiltrate the tumor engage in extensive and dynamic crosstalk with cancer cells, and several molecular events mediating this dialogue have been revealed (35). The pathogenesis of HCC is frequently linked with sustained hepatocyte death, inflammatory cell infiltration, and compensatory liver regeneration (36). In previous studies, NLR, SII, LMR, and PLR have been found can be used to predict the prognosis of patients with HCC. In diseases treated with surgical resection, transarterial chemoembolization, sorafenib or liver transplantation, pretreatment NLR^+^ is associated with poor prognosis (18, 37–39). In fact, we collected pretreatment NLR values, but some changes in NLR values may occur with tumor progression and surgical interventions, as reported in previous studies (40). The impact of neutrophils and lymphocytes on the tumor microenvironment is increasingly recognized, and prior studies have shown that large numbers of neutrophils can inhibit the activation and cytolytic anti-tumor activity of lymphocytes and natural killer cells (41). VEGF (Vascular Endothelial Growth Factor, VEGF) secreted by neutrophils in the peritumoral stroma of hepatocellular carcinoma can promote angiogenesis and ultimately tumor growth (42). Immune ratios such as NLR are predictors of microvascular infiltration in histopathology (30). In patients with HCC, these inflammatory indicators also correlate with prognosis (43, 44).

We found that combining TLSs and NLR to assess the prognosis of HCC patients is more accurate than one indicator alone. However, the small number of patients included in our current retrospective study and the different instrumentation and personnel used to test hematological indicators at each clinical research center make the test results somewhat variable. The assessment of TLSs was subjective, relying on the pathologist to evaluate it under the microscope, even though we tried to avoid such situations. Although our study has some limitations, it demonstrated the prognostic value of assessing the patient’s immune cell infiltration status from peripheral blood and peritumoral tissue together. This approach can be easily implemented in clinical practice, thus providing a paradigm for guiding and monitoring disease progression with the ultimate goal of improving the clinical outcomes of HCC patients.

## Conclusions

Both TLSs and NLR can predict the prognosis of patients with early-stage HCC. The combination of TLS density and NLR better evaluates the immune infiltrating status and predicts patient survival.

## Data Availability Statement

The raw data supporting the conclusions of this article will be made available by the authors, without undue reservation.

## Ethics Statement 

The studies involving human participants were reviewed and approved by the Ethics Committee of Jiangsu Cancer Hospital. Written informed consent for participation was not required for this study in accordance with the national legislation and the institutional requirements.

## Author Contributions

SW, BS, and YC jointly contributed the idea for this article. SW and YZC completed the manuscript, and XW made extensive revisions to the manuscript. XD, JX, and QW completed the follow-up of the data. WZ and MZ provided technical support for the staining of the sections. All authors contributed to the article and approved the submitted version.

## Funding

This work was supported by the National Science Foundation of China (Grant no: 81972313, 81972822) and Postgraduate Research and Practice Innovation Programs of Jiangsu Province (No. SJCX20_0498).

## Conflict of Interest

The authors declare that the research was conducted in the absence of any commercial or financial relationships that could be construed as a potential conflict of interest.

The reviewer, LL, has declared a shared parent affiliation with the authors, SW, YZC, CH, XD, JX, XW, QW, MZ, YC, and BS, to the handling editor.

## Publisher’s Note

All claims expressed in this article are solely those of the authors and do not necessarily represent those of their affiliated organizations, or those of the publisher, the editors and the reviewers. Any product that may be evaluated in this article, or claim that may be made by its manufacturer, is not guaranteed or endorsed by the publisher.
