# Supplementary material for: Combination of Tertiary Lymphoid Structure and Neutrophil-to-Lymphocyte Ratio Predicts Survival in Patients With Hepatocellular Carcinoma
Source: Front Immunol. 2022 Jan 13;12:788640. doi: 10.3389/fimmu.2021.788640 (PMC8793028; doi:10.3389/fimmu.2021.788640)
Supplement: Supplementary file 1 [file DataSheet_1.docx]

Supplement Figure 1


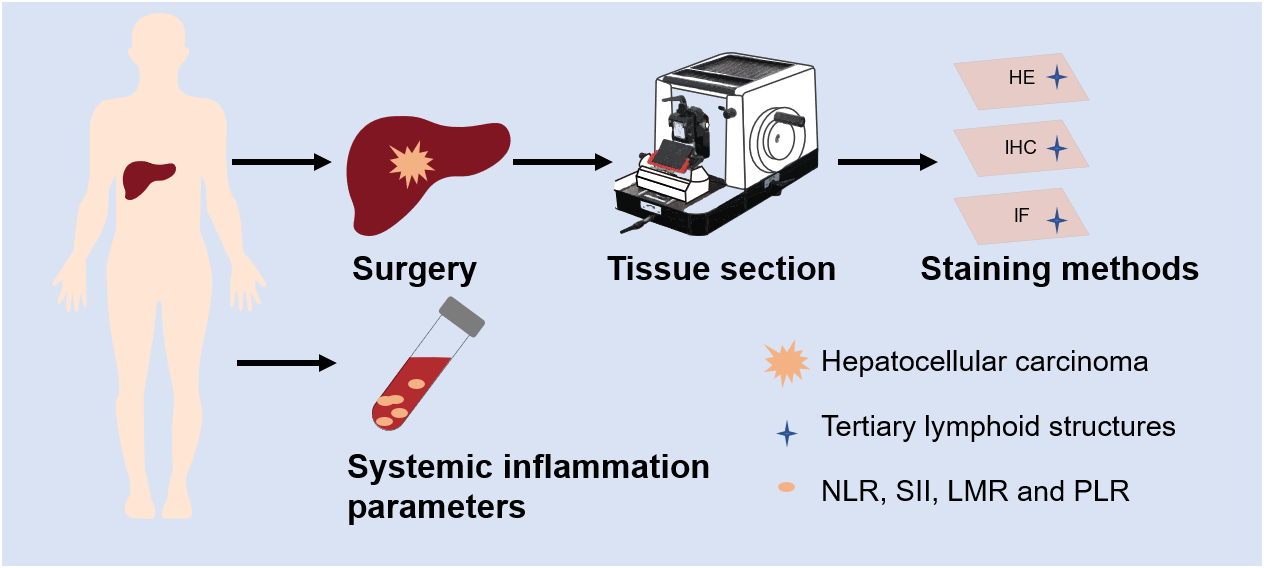


Supplement Figure 1. Tissues were obtained from hepatocellular carcinoma (HCC) patients during surgery, and liquid biopsies were performed on patients before surgery to collect several systemic inflammation parameters. Neutrophil to Lymphocyte Ratio (NLR), Systemic Immune Inflammation index (SII), Lymphocyte to Monocyte Ratio (LMR) and Platelet to Lymphocyte Ratio (PLR).

Supplement Figure 2


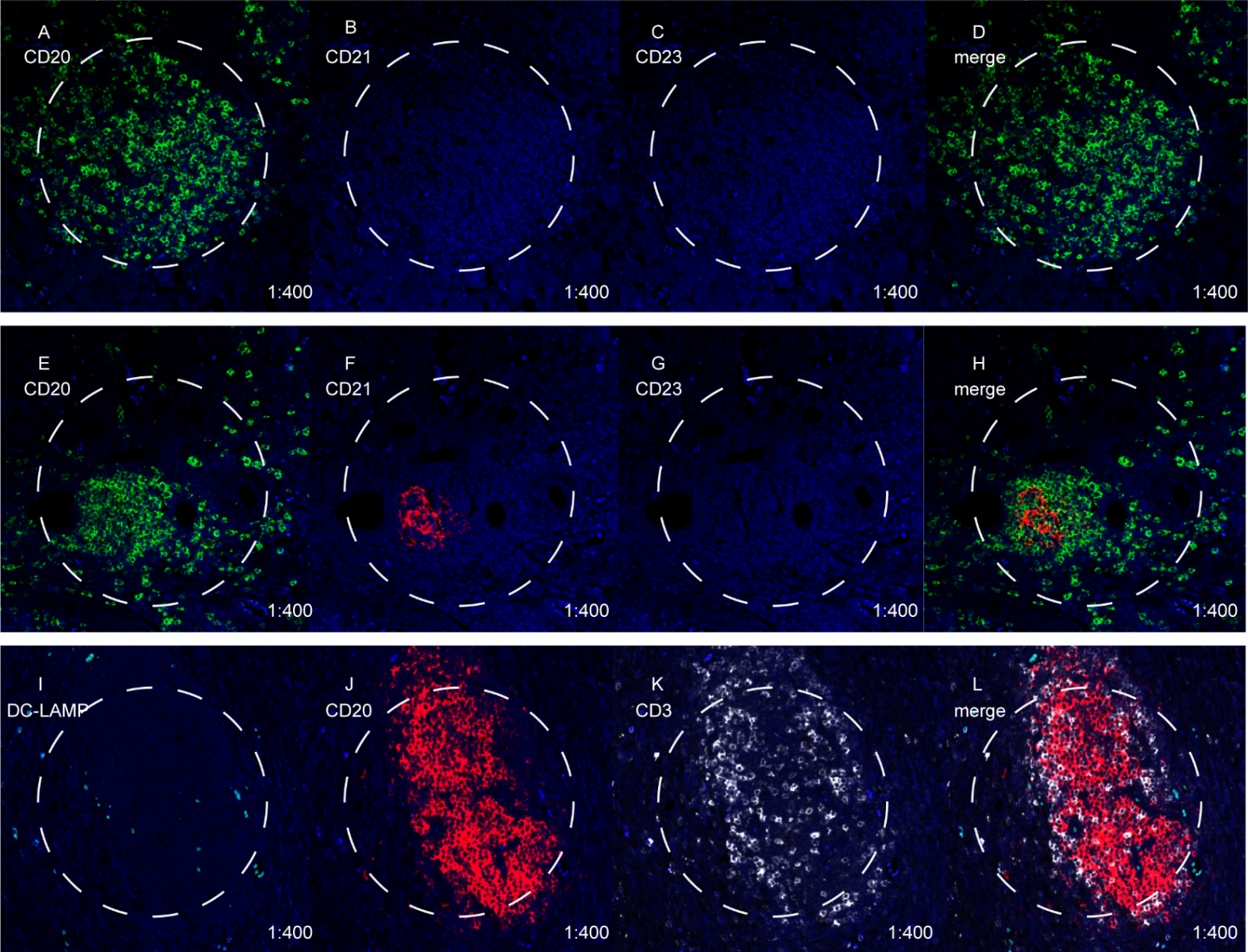


Supplement Figure 2: A-H, the co-stained immunofluorescence of CD20, CD21, and CD23, D and H is merged by CD20, CD21, and CD23’s merged. I-L, the co-stained immunofluorescence of CD20, CD3, and DC-LAMP, L is merged by CD20, CD3, and DC-Lamp’s merged.

Supplement Figure 3


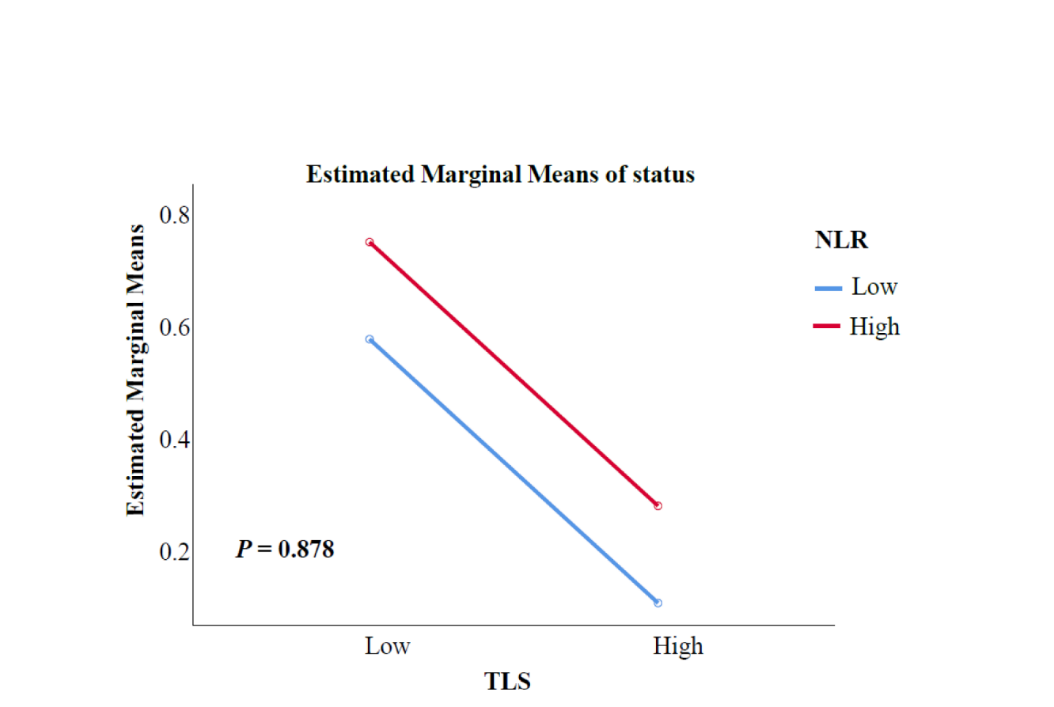


Supplement Figure 3. Interaction plot of TLS and NLR. The curves were parallel, meaning they had no interaction.

Supplement Figure 4


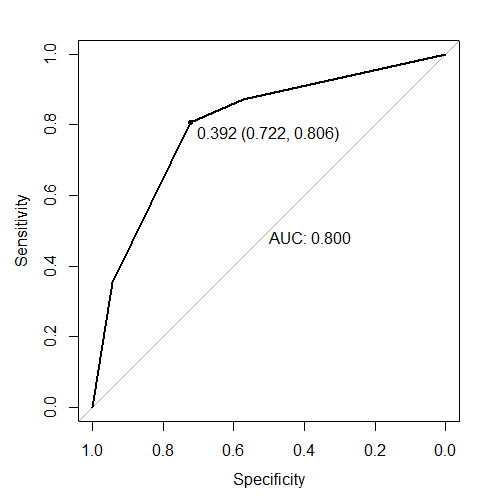


Supplement Figure 4. ROC curve for predicting the prognosis of HCC patients by combining TLS and NLR.

Supplement Table 1

|  | **Median (IQR)** |
| --- | --- |
| **Alb (g/L)** | 44.80(41.40-47.25) |
| **ALT (U/L)** | 33.10(19.85–53.65) |
| **TB (μmol/L)** | 15.20(10.85–22.15) |
| **AST (U/L)** | 31.90 (23.20–55.55) |
| **GGT (U/L)** | 57.50(31.85–122.80) |
| **APTT (s)** | 30.10(27.35–35.20) |
| **PT (s)** | 13.00(12.10–14.40) |
| **TT (s)** | 18.40(17.05–19.55) |
| **PLT (10^9/L)** | 152.00(86.00–218.00) |
| **TLS** | 0.16(0.00–0.56) |
| **NLR** | 2.56(1.69–4.03) |
| **PLR** | 103.07(77.25–148.19) |
| **LMR** | 3.06(2.13–4.09) |
| **SII** | 373.61(211.55–606.68) |

Supplement Table 1. The baseline characteristics, peripheral blood indexes, and TLS of the patients were reported as medians and interquartile ranges (IQRs) for continuous variables. Alb albumin, ALT alanine transaminase, TB Total Bilirubin, AST aspartate transaminase, GGT glutamyl transpeptidase, APTT activated partial thromboplastin time, PT prothrombin time, TT thrombin time, TLS tertiary lymphoid structures, NLR neutrophil-to-lymphocyte ratio, PLR platelet-lymphocyte ratio, LMR Lymphocyte to monocyte ratio, SII Systemic immune inflammation index.

| Supplement Table 2A Associations between clinical factors and different indicators in patients with hepatocellular carcinoma (Training set) | | | | | | |
| --- | --- | --- | --- | --- | --- | --- |
|  | **NLR High** | **NLR Low** | **P-value** | **TLS High** | **TLS Low** | **P-value** |
|  | **(N=24)** | **(N=61)** |  | **(N=45)** | **(N=40)** |  |
| **Gender** |  |  |  |  |  |  |
| man | 22 (91.7%) | 50 (82.0%) | 0.433 | 38 (84.4%) | 34 (85.0%) | 1 |
| woman | 2 (8.3%) | 11 (18.0%) |  | 7 (15.6%) | 6 (15.0%) |  |
| **Age(years)** |  |  |  |  |  |  |
| age＜54 | 14 (58.3%) | 24 (39.3%) | 0.179 | 18 (40.0%) | 20 (50.0%) | 0.48 |
| age≥54 | 10 (41.7%) | 37 (60.7%) |  | 27 (60.0%) | 20 (50.0%) |  |
| **ECOG PS** |  |  |  |  |  |  |
| 0/1 | 19 (79.2%) | 50 (82.0%) | 1 | 41 (91.1%) | 28 (70.0%) | 0.0273 |
| 2 | 5 (20.8%) | 11 (18.0%) |  | 4 (8.9%) | 12 (30.0%) |  |
| **Child Pugh class** |  |  |  |  |  |  |
| A | 18 (75.0%) | 58 (95.1%) | 0.020 | 41 (91.1%) | 35 (87.5%) | 0.852 |
| B | 6 (25.0%) | 3 (4.9%) |  | 4 (8.9%) | 5 (12.5%) |  |
| **BCLC** |  |  |  |  |  |  |
| A | 17 (70.8%) | 43 (70.5%) | 0.976 | 37 (82.2%) | 23 (57.5%) | 0.044 |
| B | 1 (4.2%) | 2 (3.3%) |  | 1 (2.2%) | 2 (5.0%) |  |
| C | 6 (25.0%) | 16 (26.2%) |  | 7 (15.6%) | 15 (37.5%) |  |
| **Alb (g/L)** |  |  |  |  |  |  |
| ＞44.20 | 13 (54.2%) | 31 (50.8%) | 0.971 | 25 (55.6%) | 19 (47.5%) | 0.600 |
| ≤44.20 | 11 (45.8%) | 30 (49.2%) |  | 20 (44.4%) | 21 (52.5%) |  |
| **Cause of hepatitis** |  |  |  |  |  |  |
| HAV | 1 (4.2%) | 1 (1.6%) | 0.798 | 1 (2.2%) | 1 (2.5%) | 0.563 |
| HBV | 17 (70.8%) | 46 (75.4%) |  | 31 (68.9%) | 32 (80.0%) |  |
| HCV | 0 (0%) | 1 (1.6%) |  | 1 (2.2%) | 0 (0%) |  |
| Unknown | 6 (25.0%) | 13 (21.3%) |  | 12 (26.7%) | 7 (17.5%) |  |
| **AFP (ng/ml)** |  |  |  |  |  |  |
| ＞37.96 | 11 (45.8%) | 32 (52.5%) | 0.757 | 22 (48.9%) | 21 (52.5%) | 0.908 |
| ≤37.96 | 13 (54.2%) | 29 (47.5%) |  | 23 (51.1%) | 19 (47.5%) |  |
| **CEA (ng/ml)** |  |  |  |  |  |  |
| ＞2.16 | 11 (45.8%) | 32 (52.5%) | 0.757 | 22 (48.9%) | 21 (52.5%) | 0.908 |
| ≤2.16 | 13 (54.2%) | 29 (47.5%) |  | 23 (51.1%) | 19 (47.5%) |  |
| **TB(μmol/L)** |  |  |  |  |  |  |
| ＞14.80 | 26 (59.1%) | 17 (41.5%) | 0.159 | 17 (37.8%) | 26 (65.0%) | 0.0221 |
| ≤14.80 | 18 (40.9%) | 24 (58.5%) |  | 28 (62.2%) | 14 (35.0%) |  |
| **PT(s)** |  |  |  |  |  |  |
| ＞13.00 | 27 (61.4%) | 17 (41.5%) | 0.106 | 18 (40.0%) | 26 (65.0%) | 0.0371 |
| ≤13.00 | 17 (38.6%) | 24 (58.5%) |  | 27 (60.0%) | 14 (35.0%) |  |
| **APTT(s)** |  |  |  |  |  |  |
| ＞30.10 | 16 (66.7%) | 28 (45.9%) | 0.138 | 17 (37.8%) | 27 (67.5%) | 0.0117 |
| ≤30.10 | 8 (33.3%) | 33 (54.1%) |  | 28 (62.2%) | 13 (32.5%) |  |
| **TT(s)** |  |  |  |  |  |  |
| ＞18.40 | 13 (54.2%) | 31 (50.8%) | 0.971 | 24 (53.3%) | 20 (50.0%) | 0.929 |
| ≤18.40 | 11 (45.8%) | 30 (49.2%) |  | 21 (46.7%) | 20 (50.0%) |  |
| **GGT(U/L)** |  |  |  |  |  |  |
| ＞52.20 | 18 (75.0%) | 25 (41.0%) | 0.0098 | 19 (42.2%) | 24 (60.0%) | 0.156 |
| ≤52.20 | 6 (25.0%) | 36 (59.0%) |  | 26 (57.8%) | 16 (40.0%) |  |
| **ALT(U/L)** |  |  |  |  |  |  |
| ＞30.00 | 19 (79.2%) | 24 (39.3%) | 0.00218 | 24 (53.3%) | 19 (47.5%) | 0.749 |
| ≤30.00 | 5 (20.8%) | 37 (60.7%) |  | 21 (46.7%) | 21 (52.5%) |  |
| **AST(U/L)** |  |  |  |  |  |  |
| ＞31.80 | 19 (79.2%) | 24 (39.3%) | 0.00218 | 22 (48.9%) | 21 (52.5%) | 0.908 |
| ≤31.80 | 5 (20.8%) | 37 (60.7%) |  | 23 (51.1%) | 19 (47.5%) |  |

Supplement Table 2A：Data were expressed as n (%) and median (interquartile range). ECOG PS Eastern Cooperative Oncology Group performance status, BCLC Barcelona clinic liver cancer, Alb albumin, HAV hepatitis A virus, HBV hepatitis B virus, HCV hepatitis C virus, AFP alpha-fetoprotein, CEA carcinoembryonic antigen, TB Total Bilirubin, PT prothrombin time, APTT activated partial thromboplastin time, TT thrombin time, GGT glutamyl transpeptidase, ALT alanine transaminase, AST aspartate transaminase.

| Supplement Table 2B Associations between clinical factors and different indicators in patients with hepatocellular carcinoma (Validation set) | | | | | | |
| --- | --- | --- | --- | --- | --- | --- |
|  | **NLR High** | **NLR Low** | **P-value** | **TLS High** | **TLS Low** | **P-value** |
|  | **(N=10)** | **(N=31)** |  | **(N=16)** | **(N=25)** |  |
| **Gender** |  |  |  |  |  |  |
| man | 8 (80.0%) | 25 (80.6%) | 1 | 14 (87.5%) | 19 (79.0%) | 0.615 |
| woman | 2 (20.0%) | 6 (19.4%) |  | 2 (12.5%) | 6 (24.0%) |  |
| **Age(years)** |  |  |  |  |  |  |
| age＜54 | 6 (60.0%) | 6 (19.4%) | 0.04 | 6 (37.5%) | 6 (24.0%) | 0.565 |
| age ≥54 | 4 (40.0%) | 25 (80.6%) |  | 10 (62.5%) | 19 (76.0%) |  |
| **ECOG PS** |  |  |  |  |  |  |
| 0/1 | 9 (90.0%) | 31 (100%) | 0.163 | 15 (93.7%) | 25 (100.0%) | 0.127 |
| 2 | 1 (10.0%) | 0 (0%) |  | 1 (6.3%) | 0 (0%) |  |
| **Child-Pugh class** |  |  |  |  |  |  |
| A | 8 (80.0%) | 31 (100%) | 0.087 | 14 (87.5%) | 25 (100.0%) | 0.285 |
| B | 2 (20.0%) | 0 (0%) |  | 2 (12.5%) | 0 (0%) |  |
| **BCLC** |  |  |  |  |  |  |
| A | 7 (70.0%) | 24 (77.4%) | 0.434 | 12 (75.0%) | 19 (76.0%) | 0.728 |
| B | 1 (10.0%) | 5 (16.1%) |  | 3 (18.8%) | 3 (12.0%) |  |
| C | 2 (20.0%) | 2 (6.5%) |  | 1 (6.3%) | 3 (12.0%) |  |
| **Alb (g/L)** |  |  |  |  |  |  |
| ＞44.20 | 8 (80.0%) | 13 (41.9%) | 0.084 | 6 (37.5%) | 15 (60.0%) | 0.278 |
| ≤ 44.20 | 2 (20.0%) | 18 (58.1%) |  | 10 (62.5%) | 10 (40.0%) |  |
| **Cause of hepatitis** |  |  |  |  |  |  |
| HAV | 0 (0%) | 0 (0%) | 0.153 | 0 (0%) | 0 (0%) | 0.714 |
| HBV | 3(30.0%) | 19 (61.3%) |  | 9 (56.3%) | 13 (52.0%) |  |
| HCV | 0 (0%) | 1 (3.2%) |  | 0 (0%) | 1 (4.0%) |  |
| Unknown | 7 (70.0%) | 11 (35.5%) |  | 7 (43.8%) | 11 (44.0%) |  |
| **AFP (ng/ml)** |  |  |  |  |  |  |
| ＞37.96 | 4 (40.0%) | 14 (45.2%) | 1 | 7 (43.8%) | 11 (44.0%) | 1 |
| ≤ 37.96 | 6 (60.0%) | 17 (54.8%) |  | 9 (56.3%) | 14 (56.0%) |  |
| **CEA (ng/ml)** |  |  |  |  |  |  |
| ＞2.16 | 6 (60.0%) | 20 (64.5%) | 1 | 8 (50.0%) | 18 (72.0%) | 0.274 |
| ≤ 2.16 | 4 (40.0%) | 11 (35.5%) |  | 8 (50.0%) | 7 (28.0%) |  |
| **TB(μmol/L)** |  |  |  |  |  |  |
| ＞14.80 | 8 (80.0%) | 11 (35.5%) | 0.037 | 6 (37.5%) | 13 (52.0%) | 0.557 |
| ≤ 14.80 | 2 (20.0%) | 20 (64.5%) |  | 10 (62.5%) | 12 (48.0%) |  |
| **PT(s)** |  |  |  |  |  |  |
| ＞13.00 | 2 (20.0%) | 7 (22.6%) | 1 | 2 (12.5%) | 7 (28.0%) | 0.434 |
| ≤ 13.00 | 8 (80.0%) | 24 (77.4%) |  | 14 (87.5%) | 18 (72.0%) |  |
| **APTT(s)** |  |  |  |  |  |  |
| ＞30.10 | 2 (20.0%) | 9 (29.0%) | 0.881 | 3 (18.8%) | 8 (32.0%) | 0.567 |
| ≤ 30.10 | 8 (80.0%) | 22 (71.0%) |  | 13 (81.3%) | 17 (68.0%) |  |
| **TT(s)** |  |  |  |  |  |  |
| ＞18.40 | 2 (20.0%) | 15 (48.4%) | 0.224 | 9 (56.3%) | 8 (32.0%) | 0.225 |
| ≤ 18.40 | 8 (80.0%) | 16 (51.6%) |  | 7 (43.7%) | 17 (68.0%) |  |
| **GGT(U/L)** |  |  |  |  |  |  |
| ＞52.20 | 6 (60.0%) | 18 (58.1%) | 1 | 12 (75.0%) | 12 (48.0%) | 0.845 |
| ≤ 52.20 | 4 (40.0%) | 13 (41.9%) |  | 4 (25.0%) | 13 (52.0%) |  |
| **ALT(U/L)** |  |  |  |  |  |  |
| ＞30.00 | 6 (60.0%) | 15 (48.4%) | 0.783 | 9 (56.3%) | 12 (48.0%) | 0.845 |
| ≤ 30.00 | 4 (40.0%) | 16 (51.6%) |  | 7 (43.7%) | 13 (52.0%) |  |
| **AST(U/L)** |  |  |  |  |  |  |
| ＞31.80 | 7 (70.0%) | 17 (54.8%) | 0.633 | 11 (68.8%) | 13 (52.0%) | 0.165 |
| ≤ 31.80 | 3 (30.0%) | 14 (45.2%) |  | 5 (31.2%) | 12 (48.0%) |  |

Supplement Table 2B：Data were expressed as n (%) and median (interquartile range). ECOG PS Eastern Cooperative Oncology Group performance status, BCLC Barcelona clinic liver cancer, Alb albumin, HAV hepatitis A virus, HBV hepatitis B virus, HCV hepatitis C virus, AFP alpha-fetoprotein, CEA carcinoembryonic antigen, TB Total Bilirubin, PT prothrombin time, APTT activated partial thromboplastin time, TT thrombin time, GGT glutamyl transpeptidase, ALT alanine transaminase, AST aspartate transaminase.
